# Sample processing for DNA chip array-based analysis of enterohemorrhagic *Escherichia coli *(EHEC)

**DOI:** 10.1186/1475-2859-7-29

**Published:** 2008-10-13

**Authors:** Pascal Basselet, Grzegorz Wegrzyn, Sven-Olof Enfors, Magdalena Gabig-Ciminska

**Affiliations:** 1 School of Biotechnology, Royal Institute of Technology (KTH), S-10691 Stockholm, Sweden; 2 Department of Molecular Biology, University of Gdansk, PL-80822 Gdansk, Poland; 3 Laboratory of Molecular Biology (affiliated with the University of Gdansk), Institute of Biochemistry and Biophysics, Polish Academy of Sciences, PL-80822 Gdansk, Poland

## Abstract

**Background:**

Exploitation of DNA-based analyses of microbial pathogens, and especially simultaneous typing of several virulence-related genes in bacteria is becoming an important objective of public health these days.

**Results:**

A procedure for sample processing for a confirmative analysis of enterohemorrhagic *Escherichia coli *(EHEC) on a single colony with DNA chip array was developed and is reported here. The protocol includes application of fragmented genomic DNA from ultrasonicated colonies. The sample processing comprises first 2.5 min of ultrasonic treatment, DNA extraction (2×), and afterwards additional 5 min ultrasonication. Thus, the total sample preparation time for a confirmative analysis of EHEC is nearly 10 min. Additionally, bioinformatic revisions were performed in order to design PCR primers and array probes specific to most conservative regions of the EHEC-associated genes. Six strains with distinct pathogenic properties were selected for this study. At last, the EHEC chip array for a parallel and simultaneous detection of genes *etpC*-*stx1*-*stx2*-*eae *was designed and examined. This should permit to sense all currently accessible variants of the selected sequences in EHEC types and subtypes.

**Conclusion:**

In order to implement the DNA chip array-based analysis for direct EHEC detection the sample processing was established in course of this work. However, this sample preparation mode may also be applied to other types of EHEC DNA-based sensing systems.

## Background

Enterohemorrhagic *Escherichia coli *(EHEC) strains comprise a subset of Shiga toxin (Verocytotoxin) – producing *E. coli *associated with serious endemic outbreaks [1-3]. They cause food-borne infections and severe, potentially fatal illnesses in humans especially among children, such as haemorrhagic colitis (HC) and haemolytic uremic syndrome (HUS) [4-6]. The infections with EHEC are often sporadic but they can also give rise to epidemics of great extent. EHEC strains that cause human infections belong to a large number of O:H serotypes. Actually, a total of 472 serotypes recovered from human infections are listed in , including more than 100 serotypes from patients with HUS [7]. Certain EHEC strains belonging to serotypes O26:H11, O103:H2, O111:H8, O145:H28, and O157:H7 have been more frequently isolated from humans with severe illnesses [8,9]. Among them, most outbreaks of HC and HUS have been attributed to strains of the enterohemorrhagic serotype O157:H7 [7]. EHEC strains of the O157:H7 serotype are the most important EHEC pathogens in North America, the United Kingdom and Japan but several other serotypes can also cause disease and are more prominent than O157:H7 in many regions in the world such as Europe, Australia, Canada, South America [10,11]. The infection source is difficult to trace because the EHEC cells are hidden among the ubiquitous non-pathogenic *E. coli*. A standard method (ISO 16654:2001) for EHEC determination is based on a confirmative analysis of the presence of the O157 antigen after a primary enrichment culture [12]. The whole procedure takes about 4 days. However, there is a low degree of correlation between the O157 presence and pathogenicity [13,14]. It was reported in the literature that many other serogroups than O157 are associated with the diseases [9,13,15,16]. There are at least two genes coding for two Shiga-toxins in *E. coli *(*stx1 *and *stx2*) [3,4,17]. Furthermore, the intimin protein, encoded by the gene *eae*, is assumed to be essential for the virulence since it accounts for the attachment of the cell to epithelial cells [18-20]. In general, the use of DNA-based analyses for identification of EHEC, rather than traditional classification in species or serological strains, offers a great advantage in the assessment of health hazards [14,21]. Here, we report on development of a method for sample processing for alternative confirmative analysis of EHEC colonies from primary enrichment cultures with the use of electric DNA chip array. The EHEC chip array for a parallel and simultaneous detection of genes *etpC*-*stx1*-*stx2*-*eae *was designed and examined. It is believed that for the assessment of *E. coli *pathogenicity, a DNA chip array with the capacity to detect the presence of the *etpC *gene, the two *stx *genes and the *eae *gene should be more efficient and rapid than the ISO method.

## Results

### Cell number count of colony

The *E. coli *strains, EDL933, CB571, 86–24, and DH5α were cultured on agar plates at 37°C for colony formation. The average diameter of the colonies was 2 ± 0.5 mm. The cell numbers in these colonies were determined by flow cytometry and evaluated against data of viable cell counting on agar plates (cfu). Both methods showed comparable values of 5 × 10^7 ^- 1 × 10^8 ^cells per colony.

### EHEC DNA preparation for chip array analysis

**Figure 1:**
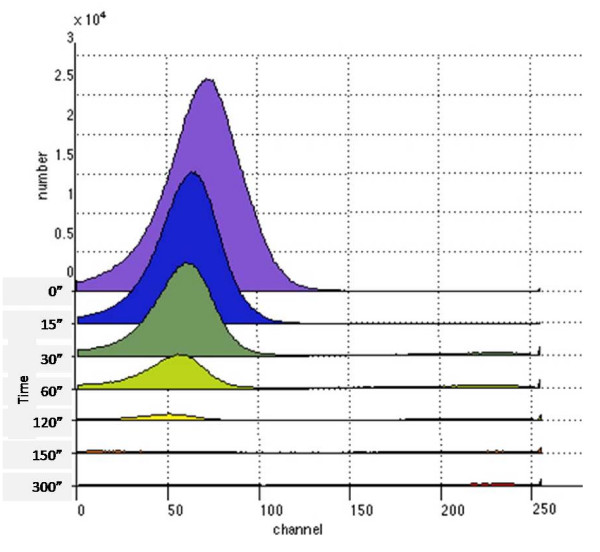
Kinetics of cell disruption by ultrasonication as shown by a histogram of forward scatter values from *E. coli *EDL933 cells subjected to 0 – 300 sec ultrasonication.

To evaluate the cell disruption during ultrasonication, samples containing 1 × 10^8 ^cells (corresponding to one agar colony) were subjected to ultrasonic disintegration followed by flow cytometry analysis (Fig. 1). The forward scatter profiles obtained for each sample are shown. At first, one broad peak with a strong signal representing non-disrupted cells was visible. With increasing ultrasonication time, this signal progressively became weaker and most of the main peak corresponding to the undisrupted cells disappeared after 150 sec sonication. Thus, the 2.5-minute sonicated sample was selected for further handling.

**Figure 2:**
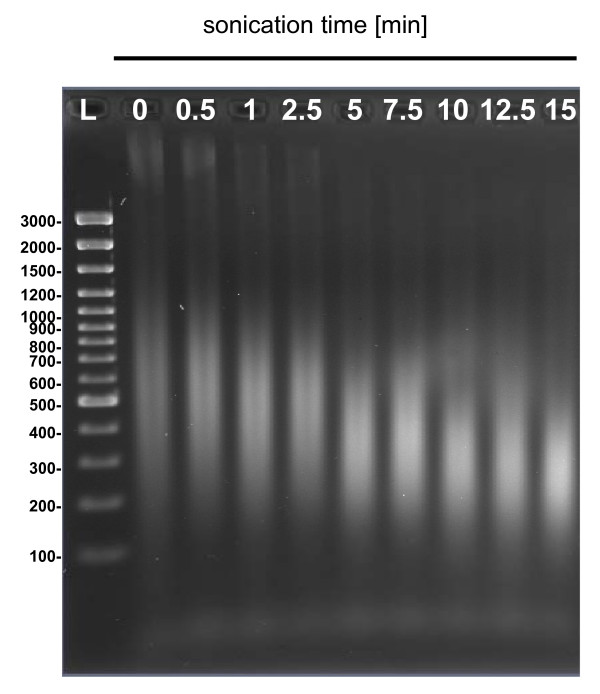
Electrophoretic analyses of distribution of genomic DNA of *E. coli *EDL933 subjected to 0 – 15 min ultrasonication. L indicates the DNA Ladder.

In the next test, the samples sonicated previously for 2.5-min were subjected to an extraction step after heat treatment and centrifugation (see Methods). DNA released was extracted two times with a phenol:chloroform:isoamyl alcohol mix, and afterwards subjected to second sonication for 0 – 15 min. Agarose gel electrophoresis was used to determine the size distribution of DNA subjected to post-extraction ultrasonic fragmentation (Fig. 2). Highly fragmented DNA was evident from the presence of a DNA smear rather than high-molecular weight bands that were eliminated from samples sonicated for 2.5 min or longer. Longer sonication gradually reduced fragment lengths to approximately 150 – 600 bp, and sonication for 15 min further degraded these fragments, as can be seen mostly by the upper part of the smear. Thus, the average DNA fragment size gradually declined with ultrasonication time and the 5 min treatment allowed to obtain the sizes of DNA fragments most suitable for chip array assays [as proved previously by 22,23]. At last, the DNA analyte preparation procedure comprising first 2 min of ultrasonic treatment, DNA extraction (2×), and subsequent 5 min sonication, was established.

### Bioinformatics study

The bioinformatics-based design was managed with all sequences of target genes (*stx1*: 74, *stx2*: 178, *eae*: 18 and *etpC*: 7) available in GenBank. The cases were subjected to a BLAST analysis against the totality of the sequences composing the database in GenBank (revised September 2007), in order to check their specificity for target genes with a threshold of e-value of 0.004. Data from literature were used as indicators for the conserved regions and were evaluated during the complete study. The PCR primers and array probes were determined by the multiple alignments of whole sequences of each virulence genes. All selected oligonucleotides are presented in details in Table 1.

**Table 1:** Oligonucleotide primers and probes used in this study.

Name	Function (Accession number)	5' position	Sequence (5' – 3')^a^
	gene *etpC *(AF401292)		
O157 Primer I	*etpC *upper primer	35621 →	ATTATGTTGTTCTTTCTATCATTCC
O157 Primer II	*etpC *lower primer	35815 ←	TTGATCACCAGTTACCGCTGTTTCC
O157 cp	*etpC *capture probe	35723 →	X - (ACCT)CTTATTGCCGGATATCAGCTGGTGT
O157 dp	*etpC *detection probe	35749 →	GGTTATCCATCATTTCTGGCTGACT(GTCA) - Y
	gene *stx1 *(M17358)		
Stx1 Primer I	*stx1 *upper primer	272 →	CGCTGAATGTCATTCGCTCTGC
Stx1 Primer II	*stx1 *lower primer	553 ←	CGTGGTATAGCTACTGTCACC
Stx1 cp	*stx1 *capture probe	406 →	X - (CTCT)GAAGGGCGGTTTAATAATCTACGGC
Stx1 dp	*stx1 *detection probe	433 →	ATTGTTGAACGAAATAATTTATATG(ACTG) - Y
	gene *stx2 *(AF500187)		
Stx2 Primer I	*stx2 *upper primer	205 →	TTTCTTCGGTATCCTATTCC
Stx2 Primer II	*stx2 *lower primer	701 ←	CTGCTGTGACAGTGACAAAACGC
Stx2 cp	*stx2 *capture probe	392 →	X - (TCTT)GCTTGATGTCTATCAGGCGCGTTTT
Stx2 dp	*stx2 *detection probe	418 →	ACCATCTTCGTCTGATTATTGAGCA(TCGG) - Y
	gene *eae *(AF022236)		
Eae Primer I	*eae *upper primer	25479 →	GGAACGGCAGAGGTTAATCTGCAG
Eae Primer II	*eae *lower primer	25805 ←	GGCGCTCATCATAGTCTTTC
Eae cp	*eae *capture probe	25568 →	X - (GCGA)GCTGGCATTTGGTCAGGTCGGAGCG
Eae dp	*eae *detection probe	25594 →	GTTACATTGACTCCCGCTTTACGGC(TTTA) - Y
NC cp	Negative Control capture probe (*acoC*)	192 →	X - ACTATCGACACGGCCCGCCTTGGAGAAGA

### Identification of virulence-related gene sequences by PCR

**Figure 3:**
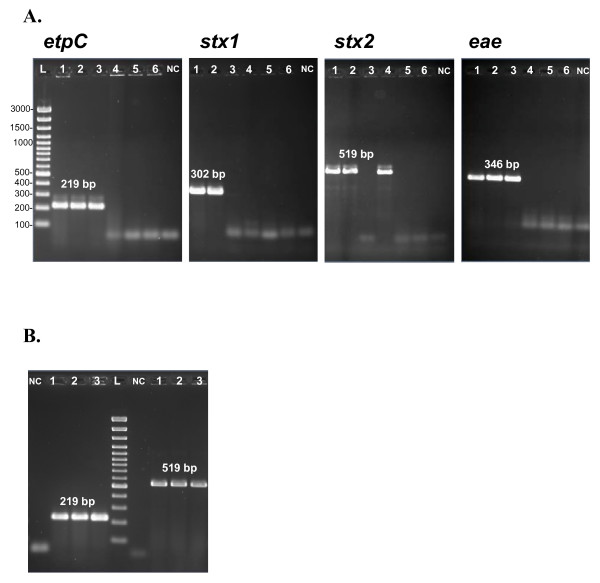
**Electrophoretic analyses of PCR amplicons: etpC (219 pb); stx1 (302 bp); stx2 (519 bp); and eae (346 bp). **A. Genomic DNAs from *E. coli *strains: (1) EDL933, (2) CB571, (3) 86–24, (4) DH5α, (5) MG1655, and (6) W3110, were applied as the PCR templates, respectively. Reaction conditions: 1-minute primer annealing at 57°C, and 2-minute elongation step. B. Genomic DNAs from *E. coli *strains: (1) EDL933, (2) CB571, (3) 86–24, (4) DH5α, were applied as the PCR templates, respectively. Reaction conditions: 2-minute primer annealing at 56°C, and 3-minute elongation step. NC is a blank PCR assay. L stands for DNA Ladder.

Genomic DNAs from all *E. coli *strains included in this study, EHEC and non-EHEC, were applied for PCR amplification of four virulence-related sequences. Fragments with expected sizes being amplicons of the *etpC*, *stx1*, *stx2 *and *eae *genes were obtained from the EDL933 and CB571 (Fig. 3A). When the *E. coli *86–24 DNA was used as a template in the PCR, predicted fragments of *etpC *and *eae *genes were amplified (Fig. 3A). Additionally, the DH5α was found to be positive for *stx2*. However, some parasitic bands for the amplification of *etpC *and *stx2 *sequences were observed. In order to improve the hybridization of primers and the work of the *Taq *polymerase, a longer time for the annealing at slightly higher temperature, and prolonged elongation was experienced. In a consequence, 2-min at 57°C, instead of 1-min at 56°C primer annealing, as well as 3- instead of 2-min elongation allowed to appreciate the optimization's effects on the PCR program with the vision of clean bands (Fig. 3B).

**Figure 4:**
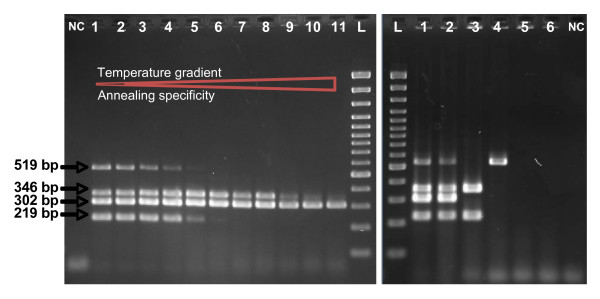
**Electrophoretic analyses of multiplex PCR amplicons: etpC (219 pb); stx1 (302 bp); stx2 (519 bp); and eae (346 bp). **A. Temperature gradient of annealing step in the multiplex PCR using *E. coli *EDL933 as DNA template. Line 1 presents the anealing temperature of 55°C; line 2: 55.3°C; line 3: 55.8°C; line 4: 56.7°C; line 5: 57.8°C; line 6: 59.3°C; line 7: 61°C; line 8: 62.4°C; line 9: 63.5°C; line 10: 64.3°C; and line 11: 65°C. For all and 3-minute elongation step was performed.B. Genomic DNAs from *E. coli *strains: (1) EDL933, (2) CB571, (3) 86–24, (4) DH5α, (5) MG1655, and (6) W3110, were applied as the PCR templates, respectively. Reaction conditions: 2-minute primer annealing at 56°C, and 3-minute elongation step. NC is a blank PCR assay. L stands for DNA Ladder.

In addition, amplification reaction conditions were determined to elaborate a multiplex PCR test allowing a quick typing of EHEC (Fig. 4). An annealing temperature gradient from 55 to 65°C was realized (Fig. 4A). No parasitic bands were seen with the increase of the annealing temperature. This analysis revealed that the 56°C as annealing temperature in the PCR assay was appropriate, so in consequence used to test all strains with the multiplex mix (Fig. 4B).

### EHEC chip array design and examination

**Figure 5:**
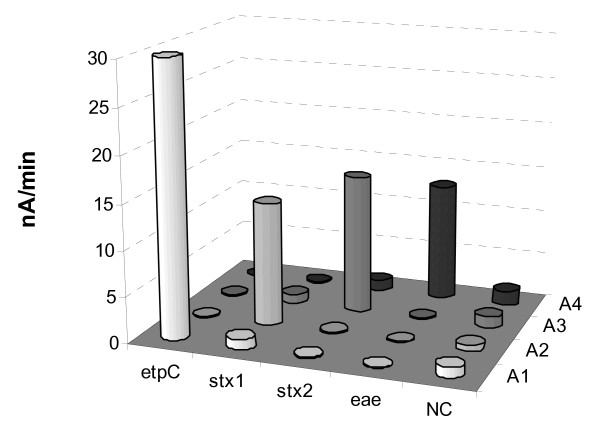
**EHEC DNA chip array assessment. **Four individual assays, A1, A2, A3 and A4, were serially conducted with four various products of simplex PCR, *etpC*, *stx1*, *stx2 *and *eae*, respectively, used as targets, and with corresponding detection probes (10 nM). 0.4 nM of each purified PCR amplicon was sequentially applied onto the chip array functionalized with capture probes. Each column is an average of three independent determinations. NC is non-biotinylated negative control capture probe of *acoC *of *B. subtilis*.

Capture oligonucleotides, i.e. O157 cp, Stx1 cp, Stx2 cp, and Eae cp (see Table 1) were designed and afterwards immobilized on randomly chosen positions of the chip array. Detection probes, i.e. O157 dp, Stx1 dp, Stx2 dp, and Eae dp (see Table 1) labeled with a biotin at the 3' end were selected to bind adjacent to the capture region of the target. Additionally, four array positions with negative control probe relevant for target sequence of *B. subtilis *were used for validation of the probes' specificity and assay performance. The EHEC chip arrays were used for hybridization assays, A1, A2, A3 and A4, with corresponding amplicons of *etpC*, *stx1*, *stx2 *and *eae*, products of simplex PCR. 0.4 nM of each purified PCR amplicon was sequentially applied to the chip test (Fig. 5). Basically, in each particular assay only specific signals were generated from the target corresponding positions. None of the spots resulted in a signal after exposure to the non-relevant PCR amplicon, indicating that no significant unspecific binding occurred. Also, no cross-reactions were observed for the negative control positions.

## Discussions

Most cases of so-called food poisoning are caused by microorganisms, among them by enterohemorrhagic *E. coli*. Routine bacteriological techniques take at least several days for detection of these bacteria or even more for their complete characterization. Since there is no correlation between serotype and pathotype, a genotypic determination is therefore necessary for the identification of these pathogenic strains. Thus, the development of EHEC detection and identification methods that cover the pathogenicity pattern is an important issue in the field of clinical diagnosis and food safety. The developments in bioinformatics of EHEC have widened the basis for its identification to include also nucleic acid analysis. As a result, new analytical techniques, monitoring devices and rapid tests have been created [21,24]. Among them, conventional and real-time PCR-based amplification methods itself or chip-based detection systems combined with bench-top PCR prior the analysis, are playing an increasingly important role [21,25,26]. Although, very sensitive, these methods remain limited for in field applications because of their inherent complexity. They are also time-consuming and offen give results problematical for data interpretation. In this respect, the most promising breakthrough in the area of rapid detection turned into platforms for real samples, is endogenous nucleic acid analysis without previous PCR. To date, only a handful of scientists worldwide have reported such analytical procedures, including optical or electrical sensing of nucleic acids isolated from cells [22,27,28]. This has a great impact on the development of rapid assays, as first prototypes of fully integrated genetic assays for sample-to-answer nucleic acid analysis already appeared, and were reported for example by Liu and co-workers [29]. The biochip device consisting of microfluidic mixers, pumps, valves, tubes and microarray sensors, allows performance of all functions including sample preparation, mixing steps, chemical reaction and electrical detection. With the use of such fully integrated biochip device the researchers reported functional detection of pathogenic bacteria from blood samples [29]. Moreover, Rudi et al. [30] developed methods of nucleic acid-based microbial sensors that cover both the sample preparation and detection approaches.

A new generation of an automatic electric chip measuring systems for the detection of pathogenic Shiga-toxin producing *E. coli *was reported lately [21]. In the most recent instrumental version added microfluidics, i.e. a complex network of valves and tubes, permits users to move liquids on and off the chip allowing for performance of multi-step assays. These miniaturized amperometric devices, based on electrical biochips made in silicon-technology, have been constructed for field applications and point of care diagnoses. An advantage of the developed system is the use of a semiconductor technology that avoids any mechanical adjustments of sensing elements, as it is necessary for optical devices. However, in this case artificial analogues such as PCR products were used as targets in the assays for biochip measurements. In the meantime, however, considerable work on the development of a procedure for bacterial sample processing for a confirmative analysis of EHEC, via simultaneous screening of virulence genes, on a single colony with DNA chip array was made. It was understood that this major technical challenge needs to be established in order to implement the technology for direct EHEC detection, and in consequence to reduce the risk of EHEC outbreaks in a rapid manner. The protocol for sample preparation includes application of fragmented genomic DNA from ultrasonicated colonies. Thus, the method requires the bacteria first to be disrupted to make the endogenous DNA available for further processing. As the extent of exposure to ultrasound increased, the formation of fragmented DNA molecules amplified. It is believed that this gives an improved diffusion-driven target movement and results in a sufficient hybridization reaction [22]. Among different methods, physical disruption is preferred, as most chemical agents inhibit the following processes, requiring removal in subsequent additional steps [31]. The use of ultrasound itself to extract DNA from cells is not a new issue, however, in this work, the procedure comprising ultrasonication implemented for the DNA fragmentation was developed and optimized with the intention to prepare samples for DNA hybridization on a chip array.

Currently, a DNA extraction step is also included in the procedure. In total, the sample preparation time for a confirmative analysis of EHEC on a single colony is app. 10 min. At the same time, the EHEC chip arrays for a parallel and simultaneous detection of genes *etpC*-*stx1*-*stx2*-*eae *were designed and examined. For this, four capture probes, each complementary to the selected target sequence, were immobilized on a 16-position chip array. Detection was realized by a biotin label placed at the 3' end of a detection probe and with the PCR products as targets. The chip arrays responded correctly with significant positive signals of the present PCR amplicons without any false positive signals.

In conclusion, the sample processing for the performance of DNA chip array-based confirmative analysis of enterohemorrhagic *Escherichia coli *(EHEC) on a single colony was demonstrated. The results obtained in the course of this work will be exploited in further study. The sample processing method will be implemented and tested in utilization of EHEC DNA chip array-based analysis performed directly on bacterial cells. It is also intended to combine the developed sample processing procedure with the chip array-based detection system in order to obtain a fully integrated unit. Less than 30 minutes will be required to perform the whole analysis. To our knowledge, this may offer the most rapid procedure for the genetic identification of EHEC described to date (commercially available GenoType EHEC enables the investigator to obtain the results of the analysis in 3 h). At last, it is worth mentioning that this sample preparation mode might also be applied to other types of EHEC DNA-based sensing systems.

## Methods

### Reagents

4-Aminophenyl β-D-galactopyranoside (pAPG), bovine serum albumin 30% solution (BSA, protease-free), ethylene-diamine-tetra-acetic acid (EDTA), ethidium bromide solution (10 mg/ml), polyoxyethylensorbitan monolaurate (Tween 20), deoxynucleotide mix (each dNTP 10 mM), *Taq *DNA polymerase (5 units/μl) and PCR buffers were purchased from Sigma-Aldrich (Steinheim, Germany). Streptavidin-β-Galactosidase conjugate (Str-β-Gal) was obtained from Roche Diagnostics Corporation (Bromma, Sweden). Agarose was bought from Boehriger (Manheim, Germany), while 6× Loading dye solution and GeneRuler™ 100 bp DNA Ladder Plus (14 bands, 100 – 3 000 bp) from Fermentas (St Leon-Rot, Germany).

All salts used for buffers were obtained from Merck KGaA (Darmstadt, Germany). Water used in all experiments was ultra-pure Milli-Q water (Millipore purification system). Phosphate-buffered saline (PBS) was prepared by dissolving 150 mM NaCl and 10 mM Na_2_HPO_4 _in water and adjusting to pH 7.4. TPBS buffer (pH 7.4) was completed by adding 0.01% (v/v) Tween 20 to PBS. DNA hybridization buffer contained 4 mM EDTA in 4× PBS solution. Working buffer was prepared by dissolving 120 mM NaCl, 30 mM K_2_HPO_4 _and 1 mM MgCl_2 _in water and adjusting to pH 7.2. Flushing buffer was prepared by adding 0.01% (v/v) Tween 20 to working buffer. Enzyme-conjugate dilution buffer was prepared by mixing 1% (v/v) BSA to flushing buffer. Tris-borate (TBE), a 0.5× solution was prepared by dissolving 0.045 M Tris(hydroxymethyl)-aminoethan-borate and 0.001 M EDTA in water and adjusting to pH 8.0. Dulbecco's buffered saline (DBS) was prepared by dissolving 160 mM sodium chloride, 3 mM potassium chloride, 8 mM disodium hydrogen phosphate dihydrate, and 1 mM potassium hydrogen phosphate dihydrate in water and adjusting to pH 7.3. All buffers were sterilized by autoclaving before use.

### Bacterial strains and cultivation

The *E. coli *strains used in this work, MG1655 (ATCC 47076), W3110 (ATCC 27325), DH5α (ATCC 53868), EDL933 (ATCC 700927), CB571 [32], and 86–24 (from Dr Gail Christie, Virginia Commonwealth University, USA) were grown aerobically in nutrient broth (LB or LA media, Merck KGaA, Darmstadt, Germany) at 37°C. Microorganisms with ATCC numbers were purchased from the American Type Culture Collection, Manassas, USA.

### Flow cytometry

Flow cytometry was realized in order to determine precisely cell counting. It was used to analyze the number of cells in isolated colonies from agar plates, and to study cell disruption by applying ultrasound to bacterial samples. All measurements were done on a Partec PAS flow cytometer (Partec, Münster, Germany) with 488 nm excitation from an argon-ion laser at 20 mW. Interferences from system noise and non-microbial particles were minimized by appropriate instrument setup, careful calibration, and filtration (0.2 μm) of all solutions prior to use. The suspended colony was further diluted 10× with DBS buffer, resulting in 1 to 2 × 10^6 ^cells/ml, which is the recommended cell density for the flow cytometry measurements. The suspension was analyzed at a flow rate of 1500–2500 counts/s. Partec Flo-Max software (version 2.4b) and MATLAB (The MathWorks, Inc) were used for data analysis and for collecting histograms of forward scatter (FSC) as a function of time. The forward scatter is considered to represent the size of cells and other measured particles [33,34].

### Arrangement of cell lysates and extracted DNAs

Bacterial pellets suspended in PBS to the desired final concentration were treated with ultrasound disruptor UP100H (Dr Hielscher GmbH, Stuttgard, Germany) equipped with a microtip 1 mm in diameter. The operating frequency was 30 kHz and effective output power was 100 W. During the operation, samples were cooled in an ice-water bath, mixed and centrifuged. The samples were utilized for flow cytometry studies, while for later handling, samples were subjected to a heat treatment (95°C, 5 min). The crude cell lysates were processed with a mixture of phenol:chloroform:isoamyl alcohol (25:24:1). An equal volume of this mix was added to the lysate sample, the solution was vortexed vigorously for 15 s and centrifuged at 15,000 x*g *for 2 min at room temperature (RT) around 22°C. The top aqueous phase containing the genomic DNA was carefully separated and collected in a new sterile Eppendorf tube.

Subsequently, samples were sonicated to fragment the DNA. The sonication step was realized in the same conditions as described above. To evaluate the fragmentation effects on the genomic DNA, samples were analyzed by using agarose gel electrophoresis.

### Design of PCR primers and probes

After selection of target genes as specific genetic markers of EHEC, primers and probes design was realized on the common areas of the sequences available in GenBank after bioinformatics analyses. The corresponding sequence accession numbers are GenBank:AF401292 (*etpC*), GenBank:M17358 (*stx1*), GenBank:AF500187 (*stx2*), and GenBank:AF022236 (*eae*). The OLIGO Primer Analysis Software, Verssion 6.88 (MedProbe, Oslo, Norway) was utilized. The upper and lower primers were designed to be single-stranded oligonucleotides of 25 nucleotides complementary to the selected region of each virulence factor representative sequence. Capture and detection probes were designed to be complementary to antisense DNA strand of each toxin-encoding sequence. An extra four nucleotides spacer (non-complementary to selected targeting sequences) at the 5' end of capture probe was added and labeled with a thiol group via a C6 chain linker, and at the 3' end of detection probe this spacer was labeled with a biotin. One negative control probe was used and described as non-biotinylated and non-relevant to any of selected four target sequences. It was designed in *Bacillus subtilis *genome (accession number: Z99108).

All oligonucleotide sequences were purchased from Thermo Electron GmbH (Ulm, Germany), and are listed in Table 1.

### PCR assays and product purification

Two types of PCRs, i.e. simplex and multiplex assays were performed in this work. Genomic DNAs used as templates for amplification of selected sequences were obtained from single colonies transferred to microtubes with a sterile loop and suspended in 50 μl of the PCR mixture. The reaction volume contained also 200 μM each of four kinds of dNTPs and 2.5 units of *Taq *polymerse in reaction buffer (10 mM Tris-HCl, 50 mM KCl, 1.5 mM MgCl_2_, pH 8.3). 0.5 μM or 0.125 μM primers were used in simplex or multiplex PCR, respectively. The amplification program consisted of 4 min of initial denaturation at 95°C, 35 cycles of 45 s of denaturation at 95°C, 1- or 2-min primer annealing at 57°C, and 2- or 3-min elongation at 72°C. The program ended with 10-min final extension step at 72°C. All PCR products were analyzed with agarose gel electrophoresis.

The purifications of the PCR amplicons were performed with Montage™ PCR Cleanup Kit from Millipore (Billerica, USA). Afterwards, 1 μl of each purified solution was analyzed by UV spectrum using a spectrophotometer ND-1000 (Nanodrop, USA). The purified PCR products' solutions were diluted to a concentration of 20 nM. Aliquots of 30 μl were stored at -20°C until their utilization for chip analyses. Purified products were additionally analyzed with agarose gel electrophoresis.

### Chip array analysis

Chip arrays with 16 electrode positions, contact pads and their connections, and the corresponding instrument 'eMicroLISA' were obtained from Fraunhofer Institute for Silicon Technology and AJ eBiochip GmbH (Itzehoe, Germany), respectively. Details of the chip array elements and instrument were described previously [35].

All electrode positions were functionalized by DNA probes via thiol-gold interaction by using a piezo electric nanodispenser NP 2.0 (GeSiM, Groβerkmannsdorf, Germany). The comprehensive protocol was provided by Elsholz et al. [35]. Each chosen capture probe was spotted on random triplicate electrode positions of the chip array. Furthermore, four negative control probe position (i.e. non- biotinylated and non-relevant to any of selected eight toxin representative sequences) were used, in order to validate detection specificity and assay performance. After functionalization, chip arrays were stored in the dry condition under the protection of nitrogen gas at RT. Before assay, single chip array was applied to the reaction chamber and flushed with 4× PBS at 50°C for 5 min.

### Assay program and detection

Purified PCR amplicons were used as target DNA analytes and mixed with detection probes (1 μM working conc. for each) in hybridization buffer. The mix (200 μl total volume) was incubated at 95°C for 5 min, then immediately put on ice for 1 min, and directly transferred in the chip reaction chamber for assay performance. The assay program was designed with steps in a sequence (Table 2). An internal iridium oxide reference electrode (+100 mV anode/-400 mV cathode) was used for all measurements.

**Table 2:** Assay program at 'eMicroLISA'

Sequence	Task	Conditions (Temperature [°C]/Time [s])
1	Wash flow	RT/20
2	Rehydration and temperature change	50/250
3	Sample transfer	50/7
4*	Hybridization	50/20
5*	Sample renewal	50/12
6	Wash flow	50/140
7	Enzyme conjugate transfer	50/6
8	Incubation	50/250
9	Wash flow	50/70
10	Temperature change	55/25
11	Substrate transfer	55/65
12	Stop flow and readout	55/35
13	Wash flow	RT/30

## Competing interests

The authors declare that they have no competing interests.

## Authors' contributions

PB performed the experiments and helped to draft the manuscript. GW edited the manuscript. SOE coordinated the study. MGC supervised the work and wrote the manuscript.
